# Data-Driven EEG Theta and Alpha Components Are Associated with Subjective Experience during Resting State

**DOI:** 10.3390/jpm12060896

**Published:** 2022-05-29

**Authors:** Povilas Tarailis, Frances M. De Blasio, Dovile Simkute, Inga Griskova-Bulanova

**Affiliations:** 1 Life Sciences Center, Institute of Biosciences, Vilnius University, Sauletekio Ave. 7, LT-10257 Vilnius, Lithuania; povilas.tarailis@gmc.vu.lt (P.T.); dovile.simkute@gmc.vu.lt (D.S.); 2 Brain & Behaviour Research Institute and School of Psychology, University of Wollongong, Wollongong, NSW 2522, Australia; francesd@uow.edu.au

**Keywords:** EEG, individual differences, subjective experience, frequency principal component analysis, f-PCA

## Abstract

The resting-state paradigm is frequently applied to study spontaneous activity of the brain in normal and clinical conditions. However, the relationship between the ongoing experience of mind wandering and the individual biological signal is still unclear. We aim to estimate associations between subjective experiences measured with the Amsterdam Resting-State Questionnaire and data-driven components of an electroencephalogram extracted by frequency principal component analysis (f-PCA). Five minutes of resting multichannel EEG was recorded in 226 participants and six EEG data-driven components were extracted—three components in the alpha range (peaking at 9, 10.5, and 11.5 Hz) and one each in the delta (peaking at 0.5 Hz), theta (peaking at 5.5 Hz) and beta (peaking at 17 Hz) ranges. Bayesian Pearson’s correlation revealed a positive association between the individual loadings of the theta component and ratings for Sleepiness (r = 0.200, BF_10_ = 7.676), while the individual loadings on one of the alpha components correlated positively with scores for Comfort (r = 0.198, BF_10_ = 7.115). Our study indicates the relevance of assessments of spontaneous thought occurring during the resting-state for the understanding of the individual intrinsic electrical brain activity.

## 1. Introduction

The resting-state paradigm is frequently applied in neuroimaging research to study spontaneous activity of the brain [1,2]. This provides a noninvasive insight into the brain’s intrinsic activity in health and disease [3,4,5,6] and helps to elucidate the role of these intrinsic activities in sensory, motor, and cognitive processes. However, despite its straightforward application, the results can be sensitive to participant-, procedure-, and measurement-related factors such as sex, emotional state, body weight, state of vigilance, participant’s body position, choice of the reference and frequency bands when the EEG is recorded, and extremal irritants such as fMRI background noise, to name a few [7,8,9,10]. While a majority of these factors can be minimized or taken into account during statistical evaluation, the participants’ mental activities, even during a brief resting-state session, cannot be precisely controlled, and the relationship between the ongoing experience of mind wandering and biological signals is still unclear [11,12,13]. Hence, there is a need to quantify participants’ subjective experiences, which can then be related to objectively observed individual physiological outcomes.

To quantify participants’ subjective experiences during a resting-state session, several resting-state questionnaires have been introduced [14,15]. The Amsterdam Resting-State Questionnaire (ARSQ) [16,17] assesses ten mind-wandering domains that participants might experience during the resting session: Discontinuity of Mind (DoM), referring to the dynamics of ongoing thoughts; Theory of Mind (ToM), referring to other-people-related thoughts; Self, referring to self-related thoughts; Planning, referring to future-directed thoughts; Sleepiness, referring to the level of drowsiness; Comfort, referring to the level of relaxation during the session; Somatic Awareness (SA), referring to the interoceptive awareness of one’s own body; Health Concern (HC), referring to general well-being; Visual Thought (Vis), referring to visual imagery during mind wandering; and Verbal Thought (VT), referring to spontaneous thoughts formulated in words. Several EEG, fMRI, and behavioral studies have reported the relationship between ARSQ domains and physiological or psychological variables in healthy [16,17,18,19,20,21,22,23,24,25,26] and clinical cohorts [27,28], confirming the importance of combined measures of subjective experience and biologically defined signals.

The quantification of subjective thoughts and emotions can help to improve the sensitivity of neuroimaging biomarkers in clinical and pharmacological studies [16]. However, the evaluation of biological signals may be performed in various ways with different purposes. For a resting-state EEG, the power spectrum is typically evaluated, and the oscillations of electrical brain activity are defined by spectral bands: delta (0.5–3.5 Hz), theta (4–7.5 Hz), alpha (8–13 Hz), beta (13.5–30 Hz), and gamma (30.5–100 Hz) [6]. Each of these traditional frequency bands is associated with distinct functions and can be affected by neuropsychiatric disorders or by vigilance state [5,6,29]. Nevertheless, the boundaries between the different frequency bands can vary across studies and labs, with some bands further being divided into sub-bands with distinct sources and functions [6,30,31], making the evaluation process biased and results difficult to generalize. Recently, attention has been paid to this problem, and methods aiming at the automatic detection of theta and alpha transition boundaries [32] or parametrizing neural power spectra as a combination of an aperiodic component and putative periodic oscillatory peaks [33] were proposed.

Alternatively, frequency principal components analysis (f-PCA) has been offered to decompose the EEG frequency spectral structure into meaningful distinct components with the inherent advantage of providing a data-driven approach across the traditional bands [31,34]. This approach has been successfully implemented in both healthy and clinical samples. Previous studies compared f-PCA outcomes in young and older subjects [35] as well as in young adults and children [36], and attributed observed differences to the effects of brain maturation. Moreover, f-PCA has been suggested as a tool to identify response biomarkers for antidepressant treatment [34,37]. The association between f-PCA outcomes and state measures has also been shown: a negative relationship was reported between multiple alpha components and skin conductance levels [38], pointing to the role of alpha activity as an index of brain arousal [39]. Finally, the components and their topographical distribution are highly similar in both eyes-open and eyes-closed conditions [31,40,41]. Nevertheless, this approach has not yet been implemented to assess the potential associations between EEG features and subjective resting-state experiences.

In this study, we implement an f-PCA approach to quantify frequency components in a large sample of young healthy volunteers, and we relate the outcomes to the subjective experiences of the resting-state condition. Based on the known associations between alpha activity and arousal, we expect at least some of the alpha-range (8–13 Hz) components (f-PCs) to be positively associated with the subjective ratings for the ARSQ domain of Sleepiness. Similarly, based on the initial observation by Diaz et al. [16], it is expected that at least one f-PC in the theta frequency range (4–7.5 Hz) is positively correlated with the ARSQ domain of Sleepiness. Finally, in line with the report by Portnova et al. [22], we anticipate that at least one of the alpha f-PCs is positively associated with the ARSQ domain of Planning. We also evaluate other possible associations between distinct f-PCs and the domains of the ARSQ.

## 2. Materials and Methods

### 2.1. Participants

Two-hundred and twenty-six participants were included in the experiment (F = 131, M = 95; age 23, 41, ±3.87). Participants’ ages ranged from 19 to 35 years. All females were healthy, nonpregnant, not using hormonal contraception, and reported experiencing regular menstrual cycles. Based on self-reports, 107 females participated in the study during the early follicular phase (menses), 20 during the luteal phase, and 4 during the ovulatory phase. All subjects gave their written informed consent to participate, and the study was approved by the Vilnius Regional Biomedical Research Ethics Committee. Participants with any reported neurological or psychiatric disorders, any kind of addiction, or the use of psychotropic substances were excluded. Participants were asked not to use nicotine and caffeine 2 h prior to the study.

### 2.2. Data Collection

Five minutes of resting-state EEG was recorded in a dim-lighted, sound-attenuated, and electrically shielded room while participants were comfortably seated in the upright position. Before the start of the recording session, participants were instructed to stay still with their eyes closed, not to think about anything in particular, and not to fall asleep. Right after the EEG recording session, participants completed the Lithuanian version of the ARSQ, where they had to retrospectively rate the statements about the emotions and thoughts from 1 (completely disagree) to 5 (completely agree).

EEG data were collected using 64 Silver/Silver Chloride electrodes placed according to the international 10–10 system and mounted on an elastic WaveGuard EEG cap and EEG equipment (ANT Neuro, The Netherlands). All electrodes were referenced against mastoids (M1 and M2) and a ground electrode was attached close to Fz. The impedance of the electrodes was kept below 20 kΩ. Two pairs of additional electrodes (VEOG and HEOG) were used. VEOG were placed above and below the right eye to record vertical eye movements, while HEOG were placed approximately 2 cm from the right and left outer canthi to record horizontal eye movements. Data were recorded with a sampling rate of 2048 Hz.

### 2.3. ARSQ

The Lithuanian version of ARSQ 2.0 was used [21]. The ARSQ is a self-report questionnaire that aims to summarize the retrospective subjective experience during a recording session. It contains 30 statements on thoughts and feelings that participants may experience during a resting-period. Each statement is rated on Likert-type scale ranging from 1 (completely disagree) to 5 (completely agree). The ARSQ covers ten different domains of resting-state cognition: Discontinuity of Mind (DoM), Theory of Mind (ToM), Self, Planning, Sleepiness, Comfort, Somatic Awareness (SA), Health Concern (HC), Visual Thought (Vis) and Verbal Thought (VT). Each domain was evaluated with three statements. The scores of each ARSQ dimension were calculated by taking the mean value of the three statements.

### 2.4. EEG Processing

The offline EEG data processing was conducted in a MATLAB (The Mathworks, Natick, MA, USA) environment using an EEGLAB toolbox [42]. The 50 Hz power line noise was removed using Thomas F-statistics implemented in the CleanLine plugin for EEGLAB [43]. EEG data were submitted to an ICA, and components with spatial and temporal characteristics of horizontal and vertical eyes movements and cardiac pulse were used to construct individual spatial filters to suppress these artefacts [44,45]. Channels with excessive artefacts were manually rejected and reconstructed using a 3D spherical spline method [46]. EEG recordings were down-sampled to 1000 Hz. Data were segmented into artefact-free, non-overlapping, 2 s epochs that were baselined over the epoch duration and decomposed using fast Fourier transform (FFT) from DC to 30 Hz with a frequency resolution of 0.5 Hz and then averaged.

### 2.5. Frequency Principal Component Analysis (f-PCA)

f-PCA analysis was conducted using an EP Toolkit [47]. A covariance matrix with unrestricted component extraction and Promax rotation was used. The cases to variables ratio was 229.7 (14012 cases: 226 participants × 62 electrodes; 61 variables: 0–30 Hz in 0.5 Hz steps). Factors with more than 3 percent of explained variance were included for further analysis, and the remaining components were excluded. To ensure a sufficient signal-to-noise ratio, electrodes with three maximum values of the component were averaged together for each distinct factor.

### 2.6. Source Localization

Standardized low-resolution electromagnetic tomography (sLORETA) [48,49] was used to determine the intracortical distribution of the electrical activity determined for f-PCs that were significantly associated with rating scores of ARSQ domains (these were set as external independent variables). The Montreal Neurologic Institute average MRI brain (MNI152) [50] was used as a realistic head model where the solution space was restricted to the cortical grey matter, corresponding to 6239 voxels at 5 × 5 × 5 mm spatial resolution. Statistical nonparametric mapping (SnMP) with 5000 permutations was used to determine the significant threshold value for voxel activation [51].

### 2.7. Statistical Analysis

Statistical analysis was performed using JASP statistical software [52,53]. The mean for each component was calculated by averaging three electrodes with the maximum values. The scores of each questionnaire domain were calculated by taking the mean value of the three statements. To evaluate the relationship between f-PCs and ARSQ domains, we used Bayesian Pearson’s correlation coefficients. Additionally, intraclass Bayesian Pearson’s correlation coefficients were calculated between all ASRQ domains. The Bayesian approach estimates the probability of a correlation for a given pair and produces a Bayes factor (BF). Bayes factors allow three different types of conclusions: evidence for alternative hypothesis (conventional significant threshold of BF > 3), evidence for null hypothesis (conventional significant threshold of BF > 1/3) and insensitive evidence (1/3 < BF < 3). The Bayesian approach does not require correction for multiple testing [54,55,56,57].

To determine the possible outliers in both f-PC and ARSQ values, a custom-written MATLAB function for multidimensional scaling (MDS) was used. MDS is a method that allows the downscaling and visualization of similarities among datasets in a low-dimensional space where the distances between datapoints optimally represent the original similarities [58,59]. MDS produced the *x* and *y* coordinates for datapoints, which were used to calculate the pairwise Euclidean distances between them. Next, the MATLAB built-in function *isoutlier* with default settings was used. The datapoints were considered outliers if they were more than three scaled median absolute deviations (MADs) away from the median. MDS was applied separately for f-PCs and ARSQ scores.

## 3. Results

Based on the MDS plot and the MAD, the final set of 226 participants did not contain any outliers either for f-PCs or for ARSQ ratings.

### 3.1. f-PCA Outcomes

**Figure 1:**
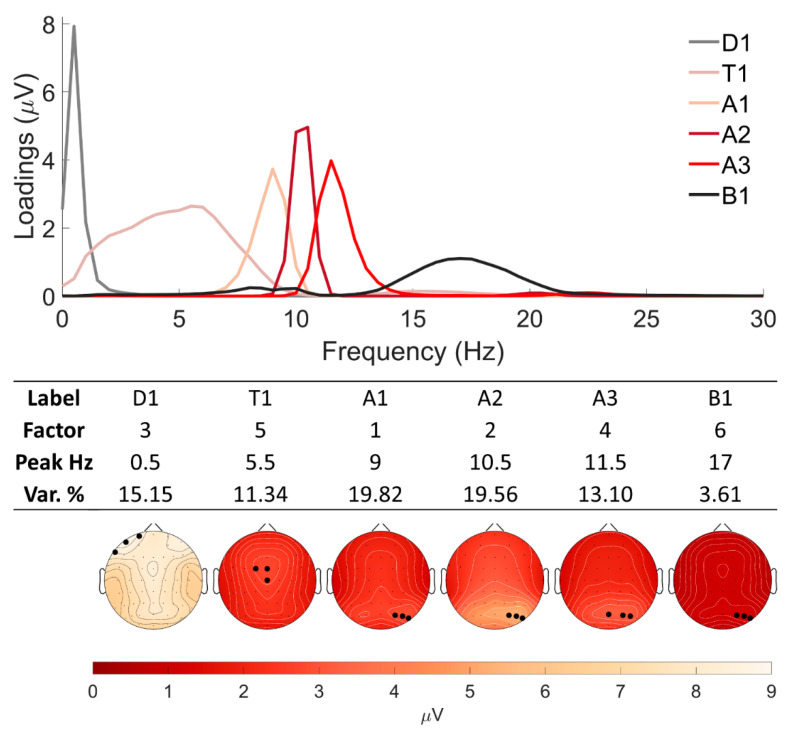
f-PCA outcomes for six factors. Top part: grand average loading scores of each factor. Middle part: individual labels, factor numbers, peak frequencies, and variance as percentage. Bottom part: grand average topographies of each component. Three electrodes with maximum values are marked with black dots.

Six factors that explained more than three percentages of variance each were used for further analysis. Together, these six factors accounted for 82.58% of the variance. The factors were ordered according to explained variance and labeled with the first letter of the corresponding EEG frequency range. There was one factor in the delta frequency range, peaking at 0.5 Hz with fronto-central activity (D1); one at the theta range, peaking at 5.5 Hz with frontal midline activity (T1); three components in the alpha range, peaking at 9 Hz (A1), 10.5 Hz (A2), and 11.5 Hz (A3), respectively, with occipital activity; and one factor in the beta frequency range, peaking at 17 Hz with occipital activity (B1). The f-PCA outcomes are depicted in Figure 1. The top part shows the loading scores of each factor, describing how much each variable contributed to a particular principal component; the middle part presents individual labels, factor numbers, peak frequencies and explained variance as percentage. The bottom part displays the topographies of each component. Three electrodes with the maximum values, which were averaged together for each component, are marked in black.

### 3.2. Subjective Reports

**Figure 2:**
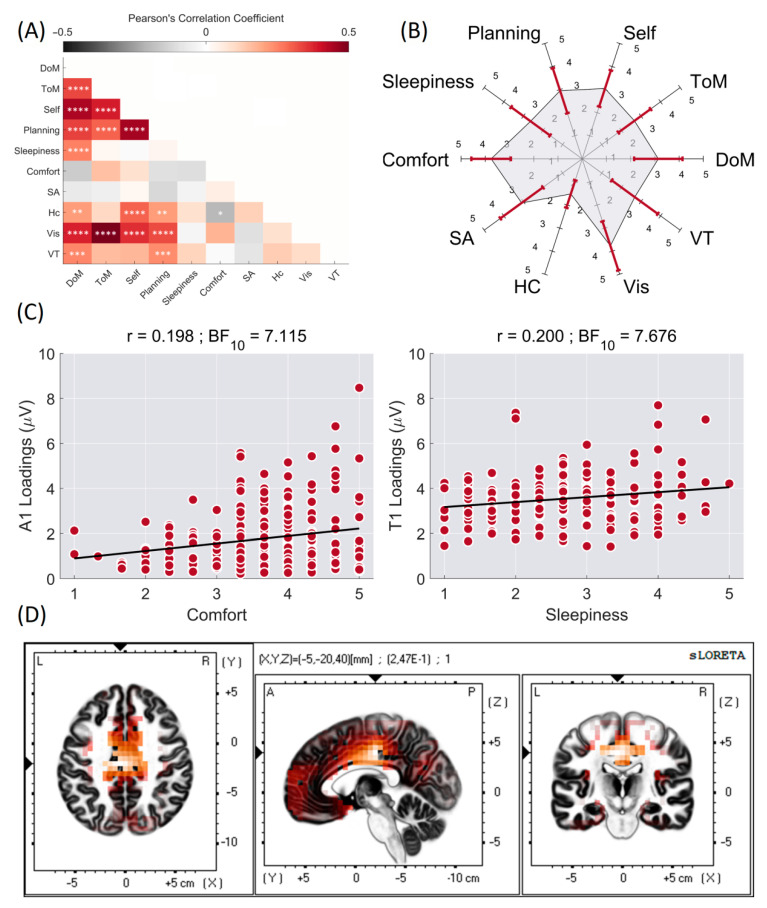
Intraclass Bayesian Pearson’s correlation coefficient between ARSQ domains. **** BF_10_ > 100, *** 100 > BF_10_ > 30, ** 30 > BF_10_ > 10, and * 10 > BF_10_ > 3 (**A**). Spider plot of mean scores and standard deviations (dark red lines) for each ARSQ domain (**B**). Scatter plots for significant interaction between activations and ARSQ domains (**C**). Intracortical activity estimated with sLORETA for T1 and ARSQ domain of Sleepiness (**D**).

The intraclass Bayesian Pearson’s correlation coefficients for the ARSQ dimensions are displayed in Figure 2A. There were, in total, sixteen positive intraclass correlations between the ARSQ domains, while only HC and Comfort displayed significantly negative relationships (r = −0.203, BF_10_ = 8.772). DoM correlated with all but the Comfort and SA domains, while SA did not display any relationship with other ARSQ domains. The mean scores and standard deviations for the scores of each ARSQ dimension are summarized in a spider plot (Figure 2B).

### 3.3. Relationship between Data-Driven EEG Components and Subjective Experiences

Out of sixty possible correlations (ten ARSQ domains x six factors) only two interactions were statistically significant (BF_10_ > 3). T1, peaking at 5.5 Hz, was positively correlated with the ARSQ domain of Sleepiness (r = 0.200, BF_10_ = 7.676). A1, peaking at 9 Hz, was positively associated with the domain of Comfort (r = 0.198, BF_10_ = 7.115) (Figure 2C). The Pearson’s correlation coefficient and BFs for correlations between the f-PC loading scores and ARSQ scores are summarized in Table 1.

### 3.4. sLORETA Results

The loadings of only two f-PCs were significantly correlated with two distinct individual ARSQ ratings. We constrained an sLORETA analysis for the A1 × Comfort and T1 × Sleepiness rating scores only.

The sLORETA analysis resulted in a significant correlation for T1 × Sleepiness (r = 0.247, *p* < 0.05), with the main activity evident in the limbic lobe, the anterior cingulate gyrus, and Brodmann areas (BAs) 24 and 23 (Figure 2D). The A1 × Comfort analysis failed to reach a significant threshold (r = 0.207, *p* > 0.05).

## 4. Discussion

The relationship between subjective experience and brain activity at rest is not well understood. An increasing number of studies have aimed at bridging the gap between objectively defined physiological signals and subjective experience during resting-state recording sessions. In this study, we focused on the association between EEG data-driven frequency components and subjective experiences measured with the Amsterdam Resting-State Questionnaire. The ARSQ appeared to be a useful tool to relate biological signals collected over the resting-state session with participants’ subjective experiences and emotions. Several studies using different brain-imaging modalities and applying different analysis methods have reported associations with the ARSQ domains (DoM [24,26], ToM [20], Self [25,26], Planning [18,22], Sleepiness [16,24], Comfort [18,24,25], Somatic Awareness [18,21,25,26], Visual Thought [24], and Verbal Thought [26]). However, none of the studies has implemented an f-PCA approach for EEG quantification.

As an outcome of f-PCA, six factors were retained: three components in the alpha range and one each in the delta, theta, and beta ranges. This is comparable with other resting-state f-PCA studies with minor differences regarding topographical display and the variances explained [31,38,40]. The f-PCA method has an advantage over traditional EEG spectral analysis, as it evaluates naturally occurring frequency components that are not affected or constrained by somewhat arbitrary, chosen EEG frequency band ranges [6,35], and it is completely data-driven.

Based on the previous observations, we expected several associations involving alpha and theta components to be present. The EEG data were mainly driven by the alpha activity; the retained three components peaking at 9, 10.5, and 11.5 Hz together explained 41.82% of the total variance. These results are in line with the results reported in other f-PCA studies, where between two [60] to five [31] components in the alpha range have been extracted, explaining from 1.4% (A2 component peaking at 9.5 Hz [40]) to 50% (alpha/theta component peaking at 9 Hz [37]) of the variance. Despite differences in the peak frequencies and explained variances, alpha-range components are topographically similar between the studies. In the current study, the A1, A2, and A3 components showed spatial correlation values ranging from 0.92 to 0.98. Functionally, alpha components were linked to the state of the subject’s arousal earlier [38]; thus, the relationship of alpha components to Sleepiness scores could be expected. However, although the retained alpha components explained almost half of the data, we observed a positive association only between A1 and the subjective ratings for the Comfort domain (r = 0.198, BF_10_ = 7.115) (Figure 2C). The Comfort domain was characterized by questions such as “I felt comfortable”, “I felt happy”, and “I felt relaxed”. Previously, this domain has been associated with the temporal characteristics of broadband EEG microstates C, E, G [25], and D [18]. EEG microstates are mainly driven by alpha activity [61]. Thus, the results, although unexpected, partially supported earlier observations. Nevertheless, the sLORETA analysis attempting to localize the relationship failed to reach a significance level for the A1 x Comfort analysis (r = 0.207, *p* < 0.05).

A single component in the theta range of T1 (peaking at 5.5 Hz) had pronounced midline frontal activity and was positively correlated with the subjective ratings for the Sleepiness domain (r = 0.200, BF_10_ = 7.676), suggesting more theta observed in subjects who reported more sleepiness. This result is in accordance with an initial report by Diaz et al. [16] evaluating the ARSQ’s relationship to physiological manifestations. The authors reported a positive correlation between the domain of Sleepiness and sustained midline theta activity. Furthermore, a positive correlation of frontal midline theta power (4–8 Hz) with subjective ratings on the Karolinska sleepiness scale was reported [62] and a negative correlation between frontal theta activity and activation of the default mode network (DMN) was shown [63]. The activity in the DMN is related both to the processing of personally significant information, self-reflection, and self-referential internal mentation [64] and to stimulus-independent thoughts [65] that are more likely to occur during a resting state. Previously, activity in the DMN was shown to positively correlate with Sleepiness [24]. Similarly to Stoffers et al. [24], we observed decisive evidence for a positive correlation between the ARSQ domains of DoM (referring to ’I had busy thoughts’, ‘I had rapidly switching thoughts’, and ‘I had difficulty holding on to my thoughts’) and Sleepiness (r = 0.251, BF_10_ = 113.972), suggesting that the more drowsiness subjects experienced, the more troubles they had in holding on to their thoughts. We attempted to localize the association between the theta component and Sleepiness and performed inverse modeling using sLORETA. The association emerged for activity in the limbic lobe and the anterior cingulate cortex (ACC) (Figure 2D). This result is compatible with reports on the localization of theta activity. Scheeringa et al. [63] reported theta activity (2–9 Hz) that originated in the medial prefrontal cortex and ACC. Smith et al. [60] localized sources of the theta component, peaking at 5 Hz, in the premotor cortex, including the dorsal ACC. Nishida et al. [66] showed theta (5–7 Hz) activation in the ACC during wakefulness and REM sleep, but not in the slow-wave sleep stage. Thus, the positive correlation between individual ratings for Sleepiness and the activity in the limbic lobe and ACC is in line with results reported in the literature.

No associations were observed with components in the delta and beta frequency ranges, although Portnova et al. [22] previously reported a negative correlation between the power spectrum density from 2–3 Hz and Planning. However, it should be noted that both the extracted D1 (peaking at 0.5 Hz) and B1 (peaking at 17 Hz) components were somewhat different from those reported previously: D1 resembled activity over the left frontal area, peaking at the FP1, AF7, and F7 electrodes, while reports in the literature have observed maximum activities in the central, fronto-central, and right frontal areas [40,41,67]. B1 had a right occipital activation with maximum values at the PO4, PO6, and PO8 electrodes that is in line with some reports [41,67] but contradicts others that showed a fronto-central topographical display [31,40]. The nature of these discrepancies is not clear. To our knowledge, our study performed an f-PCA analysis for the largest sample of young, healthy adults so far, having enough power to add to the robustness of the results.

## 5. Conclusions

In this study, we showed that (1) individual loadings of the frontal midline theta component peaking at 5.5 Hz positively correlated with subjectively experienced Sleepiness measured with the ARSQ, and (2) the individual loadings on the alpha component peaking at 9 Hz positively correlated with the subjective ratings of the Comfort domain. The observed correlations are partly in line with previously reported associations in both EEG and fMRI studies, pointing to the relevance of the assessment of spontaneous thoughts occurring during a resting state for understanding the individual intrinsic brain activity reflected in frequency principal components.

## Figures and Tables

**Table 1:** Bayesian Pearson’s correlation coefficient between six data-driven EEG components and ARSQ domains.

	Factors	A1	A2	D1	A3	T1	B1
ARSQ							
DoM	r	−0.081	−0.47	−0.008	0.009	0.059	−0.053
	BF_10_	0.173	0.107	0.084	0.084	0.122	0.114
ToM	r	0.044	0.045	0.071	0.007	0.086	0.046
	BF_10_	0.103	0.105	0.145	0.084	0.191	0.105
Self	r	−0.003	−0.062	−0.104	0.01	−0.018	0.062
	BF_10_	0.083	0.127	0.279	0.084	0.086	0.128
Planning	r	−0.018	−0.018	−0.017	−0.062	−0.048	−0.105
	BF_10_	0.086	0.086	0.086	0.128	0.107	0.288
Sleepiness	r	−0.040	−0.004	0.031	−0.052	0.200 *	−0.034
	BF_10_	0.099	0.083	0.093	0.112	7.676	0.095
Comfort	r	0.198 *	0.138	0.078	−0.044	0.131	0.082
	BF_10_	7.115	0.713	0.164	0.104	0.573	0.176
SA	r	0.027	−0.012	0.025	0.067	−0.089	−0.009
	BF_10_	0.09	0.085	0.089	0.137	0.201	0.084
HC	r	−0.008	−0.125	−0.086	−0.069	−0.025	−0.076
	BF_10_	0.084	0.475	0.189	0.142	0.089	0.158
Vis	r	0.103	0.032	−0.011	−0.116	0.128	0.1
	BF_10_	0.272	0.093	0.084	0.371	0.521	0.253
VT	r	−0.066	−0.070	−0.090	−0.044	0.02	−0.058
	BF_10_	0.135	0.145	0.206	0.103	0.087	0.121

* marks significant interactions.

## Data Availability

The data presented in this study are available on request from the corresponding author. The data are not publicly available due to privacy restrictions.
